# Disparities and burden of illness among hospitalized cancer patients with opioid dependence

**DOI:** 10.1007/s12032-026-03371-x

**Published:** 2026-09-05

**Authors:** Mariam M. Faruqui, Roberto Pili, Joel B. Epstein, Ian Lango, Poolakkad S. Satheeshkumar

**Affiliations:** 1 University at Buffalo, Buffalo, NY 14203 USA; 2 Department of Medicine, Division of Hematology and Oncology, University at Buffalo, Buffalo, NY 14203 USA; 3 City of Hope Comprehensive Cancer Center, Duarte, CA USA

**Keywords:** Disparities, Burden of illness, Opioid dependence, Cancers of the lip oral cavity and pharynx, Prostate cancers, Renal cell cancers

## Abstract

Opoid dependency (OD) is associated with adverse clinical outcomes in hospitalized patients; nevertheless, the understanding of OD and its impact on cancer patients remains restricted. Alongside addressing the symptoms related to disease, hospitalized patients with OD also grapple with mental illness (MI) challenges and the risk of infectious consequences (ICs), which remain unrecognized in cancer patients. We utilized national inpatient database to examine patient and clinical characteristics among renal cell carcinomas (RCC), prostate cancers (PC) and cancers of the lip, oral cavity, and pharynx (CLOP). We used generalized linear models to evaluate the association of OD and outcome of burden of illness (BOI), specifically length of stay (LOS). We also evaluated the association between OD and emergency department (ED) referral/admission status, ICs (specifically septicemias), weight loss, fluid and electrolyte disorders (FED), status of MI screening, and anxiety and depressive disorders. Of the 88,105 RCC there were 610 OD patients; PC (209,410) with 1115 OD; CLOP (54,265) with 495 OD. PC patients with OD were associated with longer LOS (Coefficient, 1.59; 95%CI, 1.14–2.21), higher ED status (aOR, 2.60; 1.47–4.59), higher FED (aOR, 2.25;1.59–3.19), higher septicemia (aOR, 1.61;1.09–2.39), less MI screening (aOR: 0.59, 95%CI, 0.40–0.89), higher anxiety disorders (aOR,2.31;1.53–3.48), and higher depressive disorders (aOR, 2.22; 1.48–3.33). Similarly, RCC OD patients were associated with ED status (1.66; 1.01–2.73), FED (2.40;1.50–3.83) and septicemias (2.61;1.27–2.39). These findings were comparable in the CLOP cohorts. Among all cancer cohorts, compared to Whites, Hispanics were associated with more septicemias, and Blacks, when compared to Whites, had a longer LOS. The findings are particularly significant in light of the ongoing opioid epidemic and its effects on cancer patients. In exceptional clinical situations, including a high risk of mental disorder, exact criteria must be created for controlling cancer pain that allow safe opioid delivery, taking into account biological and psychosocial OD.

**Supplementary Information:**

The online version contains supplementary material available at https://doi.org/10.1007/s12032-026-03371-x.

## Introduction

From the early phases of cancer pain treatment to palliative care in the later stages of disease, opioids are frequently required in the management for patients with moderate to severe cancer pain associated with cancer [1]. Opioids are often prescribed to help manage pain in cancer survivors with chonic lingering effects [2]. There is modest evidence of an association between opioid dependence and cancer outcomes [3], and some research even suggests opioids can cause varying degrees of health related outcomes [4]. While substance use disorders are well documented in oncology settings, opioids are still used in 34.5% of cancer treatments, albeit with careful management of risk factors, and opioids continue to be an essential component of cancer pain management [5]. Still, little is known about preexisting opioid dependence (OD) and its impact on cancer patients throughout the cancer trajectory.

Within the US, opioid use disorder prevalence was estimated to be 8%, with a 23.5% risk of use disorder for cancer-related pain according to a pooled meta-analysis [6]. It is estimated that opioid use disorder effects 2.1 million people within the US [7]. Previous studies have emphasized the relationship between cancer treatment and related pain alongside the prescription of opioids as a use of pain management. However, this has left a gap in the understanding of the relationship between cancer patients with preexisting OD and their health-related outcomes for a large group of people within the US. Both biological and psycho-social variables contribute to the development of OD, making it a complex multi-faceted condition. On the biology side, OD may be associated with cytokines and chemokines [7], sleep disturbances [8], and food deprivation [9], while psychological risk factors include psychiatric comorbidities [10], the dopaminergic reward system [11], and memory consolidation [12]. In addition to coping with cancer related symptoms, complications of cancer therapy, mental health concerns and infectious consequences are critical considerations that cancer patients must overcome [13–15].

All of these issues can lead to out-of-pocket costs and barriers to access to care. Hence, we hypothesize that cancer patients with OD are associated with worse outcomes. Opioids have been shown to have adverse effects on PC and RCC patients. For RCC, opioids can promote survival of cancerous cells [16–20], as well as decrease the number of macrophages and immune cells [21]. Opioids have also been associated with an increased recurrence of PC [22, 23], as well as migration, invasion, and growth of tumors [24]. However, opioids are also necessary as an analgesic. Cancer treatment can cause debilitating pain, and opioids are one of the essential interventions for evoking favorable pain responses [24–26].

This study aims to investigate impact of health outcomes associated with OD in prostate cancer (PC), renal cell carcinoma (RCC), and cancers of the lip, oral cavity, and pharynx (CLOP) patients, and further evalutate the associated disparities. PC is the second leading cause of cancer deaths in American men [27], and renal cell carcinoma is the most common type of kidney cancer [28]. These two cancers were chosen alongside CLOP given that smoking is a significant risk factor for all three types of cancer [29–31], and opioid dependency is also significantly associated with smoking, considering that some opioid consumption is done via smoking [32].

## Methods

### Study design and data source

The 2017 National Inpatient Sample (NIS) database, a deidentified database populated with clinical data from geographically varied U.S. institutions, was used in this retrospective cross-sectional investigation. The Healthcare Cost and Utilization Project (HCUP) of the Agency for Healthcare Research and Quality (AHRQ) provided the discharge information from the 2017 NIS database (AHRQ). The institutional IRB granted this study an exemption since it was a secondary analysis that used anonymous, de-identified data that is publicly available. With the exception of rehabilitation and long-term acute care facilities, NIS 2017 is structured as a 20% stratified sample of discharges to represent 97 percent of all inpatient hospital discharges in the United States. The NIS dataset includes information on patient sociodemographic factors, comorbidities, in-hospital outcomes, hospital attributes, and hospitalization costs.

### Study population

PC, RCC, CLOP, and OD were included in this study using ICD10-CM codes (supplementary file 1).

### Study measurements

The main independent variable was opioid dependency. Opioid Dependency was defined via ICD 10 CM code F11.20, which defines opioid dependency as “opioid dependence, uncomplicated”. In the 2017 PC, RCC, CLOP cohorts, patient- and clinical-level variables were added as covariates. The study took into account patient factors including patient location (urban/rural using a six-category urban–rural classification system), age, sex (male or female), race (White, Black, Hispanic, Asian or Pacific Islander, Native American, or Other), primary payer (Medicare, Medicaid, Private insurance, Self-pay, No charge, or Other), and primary payer based on zip code (first to fourth quartile). Clinical traits included admission origin (Transferred-in, Not-transferred), transfer type (indicator of a transfer out of the hospital), and admission type (elective vs. non-elective; elective shows whether patients were voluntarily hospitalized). Comorbidities were categorized using the Elixhauser comorbidity index. The variables for the Elixhauser comorbidity index are available in the HCUP database. Outcome variables included: BOI (length of stay), MI screening, anxiety and depression disorders, weight loss, FED, and septicemias. LOS was determined by deducting the admission date from the release date. Total charges included the cost of all healthcare services (in USD); this excludes payments to physicians and includes all hospital utilization fees levied by the hospital. In-hospital mortality was defined as patient discharge disposition codes for deaths that occurred while the patient was hospitalized (alive or dead).

### Statistical analysis

Descriptive statistics were used to describe patient and clinical characteristics stratified by race and ethnicity. To account for the complex sampling of the NIS, survey adjusted methods––survey-weighted generalized linear model (Svyglm)––were used to provide original valuations of the US population’s resulting output. The total charges and LOS were not normally distributed, so the data were log-transformed, and the geometric mean was presented. [23] A value of 0.0001 was imputed for LOS values of 0 days to avoid negative log values. For evaluating the association between OD among PC and RCC cohorts and the outcomes–LOS, anxiety and depression, MI screening, and septicemia–a Svyglm was made-to-measure. We tailored adjusted and unadjusted svyglm for LOS, total charges, in- hospital mortality, anxiety and depressive disorders, MI screening, septicemia, FED, and weight loss. Multivariable Svyglm was used to assess the risk factors associated with OD and burden of illness among cancer patients. These models were adjusted for the age, sex, payer type, patient location, race, elective, an indicator of a transfer into the hospital, median household income, and comorbidity score. For the binomial variables, we fitted a family referring quasibinomial to the Svyglm. To identify and examine patients who were hospitalized with PC or RCC or CLOP, a cross-sectional analysis was conducted. Analyses were conducted for several variables that might vary with opioid dependency. For example, those who were opioid dependent were younger than those who were not, meaning age was a significant factor. The factors found to be significant in the original analysis were adjusted for when calculating the coefficients and odds ratios of the outcome’s variables. Additional factors that were not significant in the original analysis but might affect certain outcomes variables, like comorbidities, were also adjusted for in the final analysis. The outcome variables that were not normally distributed, like LOS and total charges, outcome variables were log-transformed. Since septicemia, MI screening, ED, FED, weightloss, depressive disorders, and anxiety were dichotomous, the adjusted odds ratio was calculated with 95% confidence interval. LOS is continuous, so the coefficient was calculated. All analyses were two-tailed and statistical significance was determined using P < 0.05. All statistical analyses were performed using R 4.3.2 (R Foundation for Statistical Computing, Vienna, Austria.)

## Results

Our analysis included a total of 208,456 patients with PC, 950 of which had OD. PC patients with OD were typically younger (mean (M) = 67.62, SD = 10.01) than PC patients without OD (M = 71.48, SD = 10.81; P<0.001) (Table 1)*.* There was no statistical difference in the average total charge or mortality in PC patients with and without OD. The average LOS in days was greater in PC patients with OD (geometrical mean (*gM*) = 6.01) compared to PC patients without OD (*gM*= 4.35; P<0.001}. Additionally, PC patients with OD had more anxiety and depressive disorders, septicemia, FED and weight loss, with lower rates of MI screening*.* In the adjusted Sryglm, PC patients with OD were associated with longer LOS (Coefficient, 1.59; 95%CI, 1.14–2.21), higher ED status (aOR, 2.60; 1.47–4.59), higher FED (2.25;1.59–3.19), higher septicemia (1.61;1.09–2.39), lesser MI screening (0.59, 95%CI, 0.40–0.89), higher anxiety (2.31;1.53–3.48), and depressive disorders (2.22; 1.48–3.33).

**Table 1:** Baseline characteristics of chemotherapy induced patients with prostate cancers, with and without OD

	Prostate Cancers without OD (Weighted)	Prostate Cancers with OD (Weighted)	P- value
n	2,084,560	950	
AGE (mean (SD))	71.48 (10.81)	67.62 (10.01)	<0.001
Sex (%)			NA
Female	0.0 (0.0)	0.0 (0.0)	
RACE (%)			0.068
White	140,020.0 (69.8)	595.0 (65.4)	
Black	35,460.0 (17.7)	230.0 (25.3)	
Hispanic	14,215.0 (7.1)	55.0 (6.0)	
Asian & Others	10,995.0 (5.5)	30.0 (3.3)	
Median household income (based on current year)			<0.001
0-25th percentile	52,090.0 (25.4)	315.0 (33.3)	
26th to 50th percentile	51,775.0 (25.3)	270.0 (28.6)	
51st to 75th percentile	50,450.0 (24.6)	260.0 (27.5)	
76th to 100th percentile	50,655.0 (24.7)	100.0 (10.6)	
Expected primary payer (%)			0.001
Medicare	136,995.0 (65.8)	645.0 (67.9)	
Medicaid	9330.0 (4.5)	100.0 (10.5)	
Private insurance	53,950.0 (25.9)	175.0 (18.4)	
Self-pay, No charge and other	7850.0 (3.8)	30.0 (3.2)	
Patient Location: NCHS Urban–Rural Code (%)			0.256
"Central" counties of metro areas of >=1 million population	61,725.0 (29.7)	355.0 (37.4)	
"Fringe" counties of metro areas of >=1 million population	53,115.0 (25.6)	190.0 (20.0)	
Counties in metro areas of 250,000–999,999 population	40,240.0 (19.4)	180.0 (18.9)	
Counties in metro areas of 50,000–249,999 population	19,200.0 (9.2)	90.0 (9.5)	
Micropolitan counties and neither metropolitan or micropolitan counties	33,440.0 (16.1)	135.0 (14.2)	
Admission type (%)			<0.001
Elective	83,300.0 (40.0)	110.0 (11.6)	
Indicator of a transfer out of the hospital			0.224
Transferred out	39,300.0 (18.9)	210.0 (22.3)	
Weighted Elixir score mean (SD))	12.56 (11.86)	13.21 (11.41)	0.463
Length of Stay (Geometric mean)	4.35 days	6.01 days	<0.001
Total Charge (Geometric mean)	$ 59,484.68	$ 64,841.72	0.295
Mortality (%)	6860.0 (3.3)	40.0 (4.3)	0.459
Emergency Admissions Septicemia Anxiety Depressive Disorders Mental Illness Screenings Fed Weight Loss	102,095.0 (49.0) 21,140.0 (10.1) 14,940.0 (7.2) 16,295.0 (7.8) 57,950.0 (27.8) 56,785.0 (27.2) 20,285.0 (9.7)	750.0 (78.9) 170.0 (17.9) 175.0 (18.4) 195.0 (20.5) 175.0 (18.4) 435.0 (45.8) 200.0 (21.1)	<0.001 <0.001 <0.001 <0.001 0.005 <0.001 <0.001

All frequencies and percentages are weighted

SD, Standard deviation; NCHS, National Center for Health Statistics; $, United States’ Dollar

Our analysis also included a total of 88,105 patients with RCC, 610 of which had OD (OD). The patients with RCC and OD had a younger mean age (M = 59.35, SD = 12.04) than those with RCC and without OD (M = 65.27, SD = 13.05) (Table 2).There was no statistical difference in the average total charge, or mortality in RCC patients with and without OD. LOS was higher in RCC patients with OD (*gM* = 6.35) than RCC patients without OD (*gM*=5.06). RCC OD patients were associated with lower MI Screening (0.55; 0.32–0.94), higher ED status (1.66; 1.01–2.73), higher anxiety diorders (2.10; 1.31–3.38). higher FED (2.40;1.50–3.83), and higher septicemias (2.61;1.27–2.39).

**Table 2:** Baseline characteristics of chemotherapy induced patients with RCC, with and without OD

	RCC without OD (Weighted)	RCC with OD (Weighted)	P- value
n	87,495	610	
AGE (mean (SD))	65.27 (13.05)	59.35 (12.04)	<0.001
Sex (%)			0.67
Female	31,660.0 (36.2)	210.0 (34.4)	
RACE (%)			0.19
White	60,475.0 (71.5)	465.0 (77.5)	
Black	10,155.0 (12.0)	80.0 (13.3)	
Hispanic	8710.0 (10.3)	40.0 (6.7)	
Asian & Others	5240.0 (6.2)	15.0 (2.5)	
Median household income (based on current year)			0.149
0-25th percentile	23,700.0 (27.5)	215.0 (35.5)	
26th to 50th percentile	8185.0 (9.4)	145.0 (23.8)	
51st to 75th percentile	27,515.0 (31.5)	120.0 (19.7)	
76th to 100th percentile	18,355.0 (21.3)	85.0 (14.0)	
Expected primary payer (%)			<0.001
Medicare	47,595.0 (54.5)	320.0 (52.5)	
Medicaid	8185.0 (9.4)	145.0 (23.8)	
Private insurance	27,515.0 (31.5)	120.0 (19.7)	
Self-pay, No charge and other	4075.0 (4.7)	25.0 (4.1)	
Patient Location: NCHS Urban–Rural Code (%)			0.67
"Central" counties of metro areas of >=1 million population	24,315.0 (27.9)	210.0 (34.4)	
"Fringe" counties of metro areas of >=1 million population	21,350.0 (24.5)	125.0 (20.5)	
Counties in metro areas of 250,000–999,999 population	18,035.0 (20.7)	125.0 (20.5)	
Counties in metro areas of 50,000–249,999 population	8200.0 (9.4)	60.0 (9.8)	
Micropolitan counties and neither metropolitan or micropolitan counties	15,380.0 (17.6)	90.0 (14.8)	
Admission type (%)			<0.001
Elective	41,990.0 (48.1)	120.0 (19.7)	
Indicator of a transfer out of the hospital			0.511
Transferred out	13,750.0 (15.7)	110.0 (18.0)	
Weighted Elixir score mean (SD))	12.19 (11.69)	10.62 (12.11)	0.06
Length of Stay (Geometric mean)	5.06 days	6.35 days	<0.001
Total Charge (Geometric mean)	$ 70,113.29	$ 69,216.67	0.90
Mortality (%)	3115.0 (3.6)	35.0 (5.7)	0.19
Emergency Admissions Septicemia Anxiety Disorders Depression Disorders Mental Illness Screenings Weight Loss FED	35,440.0 (40.5) 6460.0 (7.4) 10,280.0 (11.7) 9100.0 (10.4) 22,840.0 (26.1) 9375.0 (10.7) 26,075.0 (29.8)	405.0 (66.4) 95.0 (15.6) 170.0 (27.9) 135.0 (22.1) 90.0 (14.8) 80.0 (13.1) 260.0 (42.6)	<0.001 0.001 <0.001 <0.001 0.008 0.47 0.002

All frequencies and percentages are weighted

SD, Standard deviation; NCHS, National Center for Health Statistics; $, United States’ Dollar. RCC, Renal Cell Carcinoma; OD, Opioid Dependence

Furthermore, a total of 53,770 CLOP patients were examined, with 495 of them having OD. CLOP patients with OD were typically older (M = 64.03, SD = 12.23) compared to CLOP patients without OD (M = 59.90, SD = 10.48); P<0.001 (Table 3)*.* CLOP patients with OD had higher average total charge (*gM*=$91,369) compared to those without OD (*gM* = $80,861, P<0.001). In the adjusted analyses,CLOP patients with OD were associated with increased weight loss (2.21, 1.36–3.59), increased FED (2.37, 1.49–3.77), increased anxiety disorders (1.94, 1.22–3.08), and increased LOS (1.89, 1.40–2.57).

**Table 3:** Baseline characteristics of cancers of the lip, oral cavity, and pharynx, with and without OD

	Cancers of the Lip, Oral Cavity, & Pharynx without OD (Weighted)	Cancers of the Lip, Oral Cavity, & Pharynx with OD (Weighted)	P- value
n	53,770	495	
AGE (mean (SD))	64.03 (12.23)	59.90 (10.48)	<0.001
Sex (%)			0.339
Female	15,420.0 (28.7)	165.0 (33.3)	
RACE (%)			0.674
White	38,830.0 (74.8)	380.0 (80.0)	
Black	5530.0 (10.6)	40.0 (8.4)	
Hispanic	3360.0 (6.5)	30.0 (6.3)	
Asian & Others	4215.0 (8.1)	25.0 (5.3)	
Median household income (based on current year)			0.548
0-25th percentile	14,940.0 (28.3)	165.0 (34.4)	
26th to 50th percentile	13,930.0 (26.4)	110.0 (22.9)	
51st to 75th percentile	12,665.0 (24.0)	120.0 (25.0)	
76th to 100th percentile	11,225.0 (21.3)	85.0 (17.7)	
Expected primary payer (%)			0.001
Medicare	27,020.0 (50.3)	260.0 (52.5)	
Medicaid	8210.0 (15.3)	140.0 (28.3)	
Private insurance	15,495.0 (28.9)	70.0 (14.1)	
Self-pay, No charge and other	2950.0 (5.5)	25.0 (5.1)	
Patient Location: NCHS Urban–Rural Code (%)			0.084
"Central" counties of metro areas of >=1 million population	15,405.0 (28.8)	195.0 (39.4)	
"Fringe" counties of metro areas of >=1 million population	13,785.0 (25.7)	105.0 (21.2)	
Counties in metro areas of 250,000–999,999 population	10,950.0 (20.4)	105.0 (21.2)	
Counties in metro areas of 50,000–249,999 population	5125.0 (9.6)	55.0 (11.1)	
Micropolitan counties and neither metropolitan or micropolitan counties	8295.0 (15.5)	35.0 (7.1)	
Admission type (%)			0.596
Elective	19,570.0 (36.5)	75.0 (15.2)	
Indicator of a transfer out of the hospital			<0.001
Transferred out	10,355.0 (19.3)	105.0 (21.4)	
Weighted Elixir score mean (SD))	15.73 (9.47)	12.40 (9.02)	<0.001
Length of Stay (Geometric mean)	6.44 days	8.49 days	0.063
Total Charge (Geometric mean)	$ 80,860.93	$ 91,369.23	<0.001
Mortality (%)	2335.0 (4.3)	25.0 (5.1)	0.702
Emergency Admissions Septicemia Anxiety Disorders Depression Disorders Mental Illness Screenings Weight Loss	26,200.0 (48.7) 5830.0 (10.8) 7710.0 (14.3) 6455.0 (12.0) 18,370.0 (34.2) 16,940.0 (31.5)	330.0 (66.7) 75.0 (15.2) 130.0 (26.3) 100.0 (20.2) 125.0 (25.3) 225.0 (45.5)	<0.001 0.226 0.001 0.011 0.061 0.004

All frequencies and percentages are weighted

SD, Standard deviation; NCHS, National Center for Health Statistics; $, United States’ Dollar; OD, Opioid Dependence

### Disparities in the outcomes

#### Septicemia

Among the PC OD cohort, Hispanic and Asian and other patients were more likely to have septicemia (1.27, 1.11–1.45), than White patients (1.29, 1.09–1.52). That trend was also seen in Hispanic RCC OD patients who were more likely to have septicemia (1.33, 1.09–1.62) compared to White RCC OD patients, as well as in CLOP opioid-dependent Hispanic patients(1.31, 1.03–1.67), when compared to Whites (Table 4).

**Table 4:** Multivariable Generalized Linear Model of Septicemia among PC, RCC, and CLOP patients

SEPTICEMIA	Prostate Adjusted Odds Ratio	Renal cell carcinoma Adjusted Odds Ratio	Cancers of the Lip, Oral Cavity, and Pharynx Adjusted Odds Ratio
Non-Opioid Dependent Cancer Patients	**Reference**	**Reference**	**Reference**
Opioid-dependent Cancer Patients	1.61 (1.09, 2.39)	2.16 (1.27, 3.68)	1.37 (0.70, 2.69)
Age	1.00 (1.00, 1.00)	1.00 (0.99, 1.01)	1.00 (0.99, 1.00)
Gender (Male)		**Reference**	**Reference**
Female		1.17 (1.03, 1.33)	0.98 (0.85, 1.13)
Admission type			
Non-elective	**Reference**	**Reference**	**Reference**
Elective	0.11 (0.09, 0.14)	0.18 (0.14, 0.23)	0.15 (0.12, 0.19)
Expected primary payer			
Medicare	**Reference**	**Reference**	**Reference**
Medicaid	1.00 (0.85, 1.18)	1.07 (0.85, 1.36)	0.93 (0.75, 1.15)
Private Insurance	1.02 (0.91, 1.14)	1.02 (0.85, 1.21)	0.85 (0.70, 1.03)
Self-pay & No Charge & Other	0.99 (0.81, 1.21)	1.18 (0.86, 1.62)	0.83 (0.59, 1.15)
Patient Location: NCHS Urban–Rural Code			
Central counties of metro areas of >= 1 million population	**Reference**	**Reference**	**Reference**
Fringe counties of metro areas of >= 1 million population	0.99 (0.89, 1.10)	0.87 (0.72, 1.05)	0.82 (0.68, 0.98)
Counties in metro areas of 250,000–999,999 population	0.96 (0.86, 1.08)	0.88 (0.72, 1.08)	1.06 (0.88, 1.27)
Counties in metro areas of 50,000–249,999 population	1.03 (0.88, 1.19)	0.92 (0.73, 1.08)	0.97 (0.76, 1.25)
Micropolitan counties & Not metropolitan or micropolitan counties	1.11 (0.98, 1.25)	1.05 (0.85, 1.30)	1.00 (0.80, 1.26)
Racial and Ethnic Characteristics			
White	**Reference**	**Reference**	**Reference**
Black	1.03 (0.93, 1.14)	1.21 (0.99, 1.48)	1.17 (0.96, 1.44)
Hispanic	1.27 (1.11, 1.45)	1.33 (1.09, 1.62)	1.31 (1.03, 1.67)
Asian and others	1.29 (1.09, 1.52)	1.03 (0.77, 1.36)	1.16 (0.80, 1.69)
Median household income (based on current year)			
76th to 100th percentile	**Reference**	**Reference**	**Reference**
0-25th percentile	1.01 (0.89, 1.14)	0.89 (0.72, 1.09)	0.99 (0.80, 1.21)
26th to 50th percentile	0.97 (0.87, 1.08)	0.92 (0.76, 1.12)	0.94 (0.78, 1.15)
51st to 75th percentile	1.06 (0.95, 1.18)	0.85 (0.70, 1.03)	0.96 (0.78, 1.17)
Weighted Elixir Score Mean	1.04 (1.04, 1.05)	1.06 (1.05, 1.06)	1.04 (1.03, 1.05)

Adjusted odds ratio of having septicemia for patients with opioid dependency when compared to non-dependent patients with adjusted factors

#### MI screening

PC and RCC OD patients were less likely to have had MI screening than non-OD patients (0.59, 0.40–0.89; 0.55, 0.32–0.94). PC OD patients who paid with private insurance (0.80, 0.75–0.86) and RCC OD patients who used medicaid (0.84, 0.71–0.98) were less likely to have had MI screening than those who paid with Medicare. On the other hand, PC OD patients who lived in more rural areas were more likely to have had MI screening than those living in central counties with populations over 1 million (1.11, 1.03–1.19), (1.15, 1.05–1.26), (1.15, 1.04–1.29), except for micropolitan and other counties, were the effect was not significant. Black PC (0.86, 0.80–0.93) and CLOP (0.83, 0.71–0.97) patients were less likely to have MI screening than OD White patients. Hispanic PC (0.79, 0.72–0.88), RCC (0.82, 0.71–0.94), and CLOP (0.76, 0.63–0.92) patients were less likely to have MI screening than opioid-dependent White patients (Table 5).

**Table 5:** Multivariable Generalized Linear Model of Mental Illness Screening among PC, RCC, and CLOP patients

MENTAL ILLNESS SCREENING	Prostate Adjusted Odds Ratio	Renal cell carcinoma Adjusted Odds Ratio	Cancers of the Lip, Oral Cavity, and Pharynx Adjusted Odds Ratio
Non-Opioid Dependent Cancer Patients	**Reference**	**Reference**	**Reference**
Opioid-dependent Cancer Patients	0.59 (0.40, 0.89)	0.55 (0.32, 0.94)	0.69 (0.44, 1.10)
Age	1.01 (1.01, 1.02)	1.01 (1.01, 1.02)	1.01 (1.00, 1.01)
Gender (Males)		**Reference**	**Reference**
Female		0.62 (0.57, 0.67)	0.68 (0.62, 0.75)
Admission type			
Non-elective	**Reference**	**Reference**	**Reference**
Elective	0.91 (0.85, 0.97)	1.07 (0.98, 1.17)	0.93 (0.84, 1.03)
Expected primary payer			
Medicare	**Reference**	**Reference**	**Reference**
Medicaid	0.88 (0.77, 1.00)	0.84 (0.71, 0.98)	0.90 (0.78, 1.05)
Private Insurance	0.80 (0.75, 0.86)	0.90 (0.82, 1.00)	0.97 (0.85, 1.09)
Self-pay & No Charge & Other	0.96 (0.84, 1.10)	0.95 (0.78, 1.15)	0.94 (0.77, 1.14)
Patient Location: NCHS Urban–Rural Code			
Central counties of metro areas of >= 1 million population	**Reference**	**Reference**	**Reference**
Fringe counties of metro areas of >= 1 million population	1.11 (1.03, 1.19)	1.06 (0.95, 1.19)	0.91 (0.80, 1.03)
Counties in metro areas of 250,000–999,999 population	1.15 (1.05, 1.26)	1.03 (0.91, 1.16)	1.00 (0.86, 1.15)
Counties in metro areas of 50,000–249,999 population	1.15 (1.04, 1.29)	1.07 (0.92, 1.24)	0.94 (0.80, 1.11)
Micropolitan counties & Not metropolitan or micropolitan counties	1.00 (0.92, 1.10)	0.99 (0.87, 1.13)	0.90 (0.77, 1.05)
Racial and Ethnic Characteristics			
White	**Reference**	**Reference**	**Reference**
Black	0.86 (0.80, 0.93)	0.89 (0.79, 1.01)	0.83 (0.71, 0.97)
Hispanic	0.79 (0.72, 0.88)	0.82 (0.71, 0.94)	0.76 (0.63, 0.92)
Asian and others	0.87 (0.75, 1.03)	0.83 (0.69, 1.00)	0.87 (0.74, 1.04)
Median household income (based on current year)			
76th to 100th percentile	**Reference**	**Reference**	**Reference**
0-25th percentile	0.94 (0.86, 1.03)	0.99 (0.87, 1.12)	0.94 (0.81, 1.10)
26th to 50th percentile	1.01 (0.93, 1.09)	1.04 (0.93, 1.16)	0.91 (0.78, 1.05)
51st to 75th percentile	1.03 (0.97, 1.11)	1.05 (0.94, 1.17)	0.96 (0.85, 1.09)
Weighted Elixir Score Mean	1.00 (1.00, 1.01)	1.00 (1.00, 1.01)	1.00 (0.99, 1.00)

Adjusted odds ratio of having had mental illness screening for patients with opioid dependency when compared to non-dependent patients with adjusted factors

#### LOS

Overall, OD PC and CLOP patients were more likely to have longer LOS than non-OD patients (Coeff 1.59, 95% CI 1.14 − 2.21, Coeff 1.89, 95% CI 1.40–2.57). PC OD patients with Medicaid had a longer LOS, when compared to those who used Medicare (1.18, 1.05–1.33). PC OD patients with private insurance or self-pay, no charge, or other had shorter LOS than those on Medicare (0.91, 0.86–0.97), (0.75, 0.64–0.88), as did those who had elective admissions (0.69, 0.63–0.75). Black PC, RCC, and CLOP patients who had OD had significantly longer LOS than Whites (1.20, 1.12–1.28), (1.21, 1.12–1.28), (1.29, 1.11–1.51), but there was no difference between Hispanics and Asians and others versus Whites, except for CLOP patients where Asian and others saw increased LOS (1.22, 1.03–1.43). PC and CLOP OD patients in low-income neighborhoods (zip codes) had longer LOS than those in high-income neighborhoods (1.10, 1.03–1.18), (1.33, 1.13–1.57). PC, RCC, and CLOP opioid-dependent patients with more comorbidities had longer LOS (1.04, 1.04–1.05), (1.04, 1.03–1.04), (1.05, 1.04–1.05) (Table 6).

**Table 6:** Multivariable Generalized linear model of Length of Stay among PC, RCC, and CLOP patients

LENGTH OF STAY	Prostate Cancer Coefficient	Renal cell carcinoma Coefficient	Cancers of the Lip, Oral Cavity, and Pharynx Coefficient
Non-Opioid Dependent Cancer Patients	**Reference**	**Reference**	**Reference**
Opioid-dependent Cancer Patients	1.59 (1.14, 2.21)	1.22 (0.75, 1.99)	1.89 (1.40, 2.57)
Age	1.01 (1.00, 1.01)	1.00 (1.00, 1.00)	1.00 (1.00, 1.00)
Gender (Males)		**Reference**	**Reference**
Female		1.08 (1.01, 1.14)	0.96 (0.87, 1.07)
Admission type			
Non-elective	**Reference**	**Reference**	**Reference**
Elective	0.69 (0.63, 0.75)	1.02 (0.95, 1.11)	1.21 (1.09, 1.35)
Expected primary payer			
Medicare	**Reference**	**Reference**	**Reference**
Medicaid	1.18 (1.05, 1.33)	1.08 (0.95, 1.22)	1.22 (1.03, 1.44)
Private Insurance	0.91 (0.86, 0.97)	0.94 (0.87, 1.01)	1.06 (0.93, 1.21)
Self-pay & No Charge & Other	0.75 (0.64, 0.88)	0.93 (0.78, 1.12)	0.99 (0.76, 1.28)
Patient Location: NCHS Urban–Rural Code			
Central counties of metro areas of >= 1 million population	**Reference**	**Reference**	**Reference**
Fringe counties of metro areas of >= 1 million population	1.04 (0.97, 1.11)	1.07 (0.98, 1.17)	1.06 (0.91, 1.22)
Counties in metro areas of 250,000–999,999 population	1.06 (0.99, 1.13)	1.04 (0.95, 1.14)	1.11 (0.97, 1.27)
Counties in metro areas of 50,000–249,999 population	1.07 (0.98, 1.16)	1.06 (0.95, 1.18)	1.03 (0.86, 1.24)
Micropolitan counties & Not metropolitan or micropolitan counties	0.99 (0.91, 1.08)	1.02 (0.93, 1.12)	0.89 (0.75, 1.04)
Racial and Ethnic Characteristics			
White	**Reference**	**Reference**	**Reference**
Black	1.20 (1.12, 1.28)	1.21 (1.12, 1.28)	1.29 (1.11, 1.51)
Hispanic	1.02 (0.92, 1.2)	0.94 (0.92, 1.24)	1.12 (0.92, 1.37)
Asian and others	1.06 (0.94, 1.18)	1.04 (0.94, 1.18)	1.22 (1.03, 1.43)
Median household income (based on current year)			
76th to 100th percentile	**Reference**	**Reference**	**Reference**
0-25th percentile	1.10 (1.03, 1.18)	1.09 (0.99, 1.20)	1.33 (1.13, 1.57)
26th to 50th percentile	1.04 (0.97, 1.11)	1.09 (1.00, 1.19)	1.21 (1.03, 1.43)
51st to 75th percentile	1.02 (0.96, 1.09)	1.07 (0.98, 1.17)	1.06 (0.92, 1.23)
Weighted Elixir Score Mean	1.04 (1.04, 1.05)	1.04 (1.03, 1.04)	1.05 (1.04, 1.05)

Coefficient of length of stay for patients with opioid dependency when compared to non-dependent patients with adjusted factors

#### Depression

PC OD patients were more likely to have depression than non-OD patients (2.22, 1.48–3.33). PC and CLOP OD patients with elective admissions were less likely to have depression (0.42, 0.38–0.48), (0.65, 0.56–0.75). PC and CLOP OD patients with private insurance (0.64, 0.57–0.73) and CLOP patients who self-paid (0.58, 0.41–0.82) were less likely to have depression when compared with Medicare. Black, Hispanic and Asian and Other PC (0.58, 0.51–0.66) (0.62, 0.52–0.74) (0.57, 0.47–0.69) and CLOP patients (0.66, 0.52–0.83) (0.74, 0.56–0.97) (0.48, 0.36–0.65) were less likely to have depression than OD White patients. Further, Hispanic RCC OD patients were less likely to have depression compared to White RCC OD patients (0.82, 0.71–0.95) (supplementary file 2).

#### Anxiety

PC, RCC, and CLOP OD patients were more likely to have anxiety than non-OD patients (2.31, 1.53–3.48; 2.10, 1.31–3.38; 1.94, 1.22–3.08). Among all cancers, OD elective admissions were less likely to have anxiety than non-elective admissions (0.61, 0.54–0.68) (0.79, 0.70–0.90) (0.83, 0.73–0.94). PC and RCC, OD patients with private insurance (0.70, 0.62–0.78) (0.77, 0.67–0.89) as well as RCC OD patients with self-pay (0.74, 0.58–0.96) were less likely to have anxiety than those with Medicare. Black, Hispanic, Asian,f and Others PC OD (0.43, 0.34–0.49) (0.59, 0.49–0.70) (0.64, 0.50–0.81), RCC OD (0.49, 0.40–0.59) (0.51, 0.42–0.62) (0.54, 0.40–0.74), and CLOP OD patients (0.44, 0.35–0.56) (0.66, 0.51–0.86) (0.55, 0.38–0.78) were less likely to have anxiety than OD White patients (supplementary file 3).

#### FED

PC, RCC, and CLOP opioid-dependent patients were more likely to have FED (Fluid and Electrolyte Imbalance) than non-opioid dependent patients (aOR 2.25, 95%Ci 1.59–3.19, aOR 2.40, 95%CI 1.50–3.83, aOR 2.37, 95%CI 1.49–3.77). For RCC and CLOP opioid-dependent patients, females were more likely than males to have a higher FED (1.36, 1.25–1.48), (1.27, 1.14–1.42). Elective admissions were less likely to have FED for PC, RCC, and CLOP patients (0.39, 0.35–0.42), (0.60, 0.54–0.66), (0.31, 0.27–0.34). There were no significant differences among different expected primary payers or patient location. Black PC opioid-dependent patients were associated with higher FED than white patients (1.14, 1.05–1.23). PC, RCC, and CLOP opioid-dependent patients with higher comorbidities were associated with higher FED (1.11, 1.11–1.11), (1.11, 1.11–1.12), (1.10, 1.10–1.11) (supplementary file 4).

#### Weight loss

PC and CLOP OD patients were associated with higher weight loss compared to non-OD patients (2.96, 1.81–4.83; 2.21, 1.36–3.59). For OD RCC patients, females were associated with higher weight loss compared to males (1.18, 1.05–1.33). PC, RCC, and CLOP OD patients with elective admissions were associated with decresed weight loss (0.49, 0.42–0.57), (0.53, 0.45–0.62), (0.52, 0.46–0.59). PC, RCC, and CLOP opioid-dependent patients with Medicaid (1.20, 1.01–1.43), (1.59, 1.29–1.97), (1.49, 1.26–1.76), and RCC self-pay OD patients (1.44, 1.09–1.89) were associated with increased weight loss compared to Medicare. On the other hand, PC OD patients with private insurance were associated with decreased weight loss (0.85, 0.75–0.96). PC opioid-dependent patients in counties in metro areas of 250,000–999,999 population (0.87, 0.77–0.99) were associated with decreased weight loss compared to central counties of metro areas of >=1 million population. RCC OD patients in low, low-mid, and mid-high income neighborhoods were associated with increased weight loss compared to those in high income neighborhoods. CLOP OD patients in low income and low-mid income neighborhoods were associated with increased weight loss compared to those in high income neighborhoods (1.20, 1.02–1.40), (1.32, 1.12–1.54) (supplementary file 5).

#### Emergency admissions

PC and RCC OD patients were associated with increased ED compared to non-OD admissions (2.60, 1.47- 4.59; 1.66, 1.01–2.73). RCC and CLOP OD females patients had higher associations with ED admission compared to male patients (1.12, 1.01–1.25). PC, RCC, and CLOP OD patients with Private insurance had lower ED status than those with Medicare (0.81, 0.72–0.92), (0.86, 0.74–0.99), (0.78, 0.66–0.92). PC, RCC, and CLOP patients living in micropolitian counties and not metropolitan or micropolitan counties were associated with decreased ED compared to those living in central counties of metro areas of >= 1 million population (0.42, 0.35–0.51), (0.46, 0.37–0.57), (0.67, 0.53–0.85). Hispanic PC and CLOP OD patients had higher associations of ED compared to White patients (1.49, 1.24–1.80), (1.63, 1.25–2.12). PC OD patients in the low, low-mid, and mid-high median household income neighborhoods (0.84, 0.71–0.98) (0.76, 0.66–0.88) (0.82, 0.72–0.94), and CLOP OD low-mid median household income neighborhoods (0.81, 0.66–0.97) had lower ED status compared to those in high income neighborhoods (supplementary file 6).

## Discussion

This study of hospitalized PC, CLOP, and RCC patients using the inpatient database found that having OD was associated with disparities associated with outcomes: higher rates of septicemia for PC and RCC patients, higher rates of depressive disorders for PC patients, and higher rates of anxiety for PC, RCC, and CLOP patients. There was a greater burden of illness (LOS) for PC, RCC, and CLOP patients. Having OD was also associated with lesser mental health screening, while CLOP, PC, and RCC patients with OD was also associated with mental health diorders (anxiety disorders). These findings should be recognized for their demonstrable impact upon patient care.

There is significant literature investigating the characteristics of OD patients. One study found that there were high rates of both global and domain-specific neurocognitive impairments and OD adults, with specifically severe impairments in learning and memory [33]. Additionally, lifetime alcohol and cocaine dependence were associated with greater impairment for those with OD [33]. Studies also reported that those who smoke are more likely to being misusing opioids when compared to those who have never smoked [32]. Race, region, income, age, gender, depression or antidepressant use, and health insurance are all risk factors for OD under treatment of pain [34]. In previous studies in North Carolina, patients with OD were younger, disproportionately White, had lower incomes, and were self-payers for insurance [35]. The outcomes of the OD investigation exhibit variability based on race, social class, and gender, with participants evaluating working-class opioid users more critically than their middle-class counterparts, and White users more severely than Black users. Literature also indicate middle-class participants were more likely to have OD when compared to lower-class participants, although this may interact with the other factors [36]. On the other hand, having more resources for OD management is associated with favorable outcomes [37]. Among patients transitioning from homeless living to housed environments, Whites responded best to treatment for OD [38], which may include effective management of opioid withdrawal symptoms [39] or opioid agonist treatment [40].

OD has also been shown to be associated with clinical outcomes. In one study, the most common co-occurring diagnoses were psychoses, substance abuse or dependence, and septicemia [35]. However, this may be due to differences in rates of being diagnosed since White patients have greater odds of being diagnosed with opioid use disorder in a Medicaid-managed population [41]. There is evidence of a causal relationship between being genetically inclined towards OD and risk of major depressive disorder and stress-related disorders [42]. For those with chronic pain managed by opioids, post-traumatic stress is associated with increased anxiety sensitivity [43] and pain-related anxiety [44], which is associated with opioid misuse and dependence [45], and this effect is cumulative [43]. Studies suggest that anxiety sensitivity is associated with motives for pain management and coping when it comes to opioid misuse, and not for variance of pain intensity [46]. Negative mood, a symptom of both anxiety and depression, may be the sub facet of both conditions that leads to opioid abuse [37], although racial and income based information are unknown. For those with depression, impaired memory, perceived stress, and low mood are the main symptom subgroups that are risk factors for OD [47]. Pain-related anxiety also modulates the effect of tobacco use on OD [48]. OD also has implications in prescription for pain management, requiring higher dose of opioid for pain management and close follow-up, as well as co-analgesic medications and alternative pain management approaches.

Additionally, OD is specifically associated with worse clinical outcomes related to burden of illness for a variety of conditions. Opioid-dependent women have longer LOS [49]. In general, for all patients in the intensive care unit, OD was associated with mortality and increased LOS [50]. For patients receiving urological oncological surgery, OD was associated with higher rates of mortality, increased LOS, and higher inpatient costs [51]. For those in the hospital after major abdominal surgery, OD was associated with both major complications and increased LOS [52]. In a study conducted in South Australia, general surgery patients with oxycodone allergies who were prescribed opioids for pain management had higher overall costs [53]. Among traumatic brain injury (TBI) patients, those who abused opioid prior to being admitted had lower mortality and myocardial infarction but higher risks of respiratory and neurological complications [54]. They were also more likely to have a host of co-morbidities, including sepsis, malnutrition, delirium, respiratory failure, and renal failure [54]. Interestingly, TBI patients who abused opioids prior to admission had shorter LOS in the hospital when compared to TBI patients who did not abuse opioids [54].

As shown through the disparities in the OD outcomes, the social determinants of health may interact with OD to impact clinical outcomes. Among OD patients, Hispanic patients, those on Medicare, and people living in high-income areas had longer LOS [35]. On the other hand, areas of low socioeconomic status have higher overdose mortality rates associated with OD [55]. Septicemia is one of the most common co-occurring diagnoses with OD [35, 56], and that number has been rising in recent years as the opioid crisis has worsened [56] and is of major concern. Septic patients with opioid-related hospitalizations were younger with fewer comorbidities and lower mortality, even though they had more bloodstream infections from bacterial and fungal pathogens [40]. Among those who died of sepsis, a significant proportion were hospitalized for opioid-related issues [56]. This was especially true for patients under fifty [56]. There are also unusual cases of opioid-dependent patients who develop severe sepsis [57]. The risk is particularly high for pregnant women: pregnant women with sickle cell disease were four times more likely to have opioid-related disorders than those without sickle cell disease, which makes sense since opioids are commonly prescribed to manage the pain [58]. Pregnant women with sickle cell disease and related opioid-use disorder had an increased risk of maternal sepsis, which threatened preterm labor and discouraged fetal growth [58].

OD is a serious problem in the United States. While opioids are necessary for pain management in cancer patients, there is some evidence they may facilitate cancer growth and lead to proliferation of tumors [16–24]. This study reaffirmed that opioids are associated with the social determinants of health, including race, income, gender, insurance payer, and other patient characteristics. OD was also associated with a host of co-morbidities, like anxiety, depression, and septicemia, and opioid dependent adults are more likely to have worse clinical outcomes, from general problems like mortality and major complications after surgery, to outcomes associated with burden of illness, like length of stay and total charges. These clinical outcomes are affected by the interaction of the social determinants of health and opioid dependency such that those with risk factors for OD like low income are not only more likely to be opioid-dependent, they are also more likely to have worse clinical outcomes for various conditions, including PC and RCC, if they are opioid-dependent. This holds especially true for septicemia, which is often co-diagnosed with opioid dependency [56].

## Limitations

The findings of the study must be recognized within the framework of these limitations. Due to discharges, a single patient may be readmitted and subsequently counted multiple times. However, this is very unlikely, as that information should be in the readmission database. The data set also lacked information on cancer stage and tumor size, as well as specific regimens or cycles, which precluded the considerations of the effects of these variables in our analyses. Additionally, ICD-10 codes were utilized to identify OD, which may be prone to coding errors. This was addressed via validation through various cancer cohorts as well as inclusion and exclusion criteria that altogether support the adequacy of our findings based on ICD CM codes. Finally, all analyses were conducted using a national inpatient database, which limits the interpretation of the findings due to residual confounding and lack of temporal sequencing. Further research could involve validation in an independent cohort such as a separate registry or institutional dataset in order to strengthen the findings of this study.

## Conclusion

The availability of opioids is crucial in order to provide high quality care for this specific patient population. However, adequate care and resources are needed in order to help those who are afflicted by opioid dependency. A lack of those resources is associated with the disparities seen in this study. An increase in resources and care for opioid dependent cancer patients could be critical in improving patient health outcomes for the most vulnerable populations.

## Supplementary Information

Below is the link to the electronic supplementary material.

Supplementary file1 (DOCX 19 kb)

Supplementary file2 (DOCX 18 kb)

Supplementary file3 (DOCX 18 kb)

Supplementary file4 (DOCX 17 kb)

Supplementary file5 (DOCX 17 kb)

Supplementary file6 (DOCX 17 kb)

## Data Availability

No datasets were generated or analysed during the current study.
