# Supplementary material for: Disparities and burden of illness among hospitalized cancer patients with opioid dependence
Source: Med Oncol. 2026 Sep 5;43(10):267. doi: 10.1007/s12032-026-03371-x (PMC13546320; doi:10.1007/s12032-026-03371-x)
Supplement: Supplementary file 1 [file 12032_2026_3371_MOESM1_ESM.docx]

**Supplementary file 1**

**ICD 10 CM billable Codes.**

**Prostate.**

**C61 Malignant neoplasm of prostate**

**Cancers of the lip, oral cavity and pharynx**

(C00-C14).

- [C00.0](https://icdlist.com/icd-10/C00.0) - Malignant neoplasm of external upper lip **BILLABLE CODE**
- [C00.1](https://icdlist.com/icd-10/C00.1) - Malignant neoplasm of external lower lip **BILLABLE CODE**
- [C00.2](https://icdlist.com/icd-10/C00.2) - Malignant neoplasm of external lip, unspecified **BILLABLE CODE**
- [C00.3](https://icdlist.com/icd-10/C00.3) - Malignant neoplasm of upper lip, inner aspect **BILLABLE CODE**
- [C00.4](https://icdlist.com/icd-10/C00.4) - Malignant neoplasm of lower lip, inner aspect **BILLABLE CODE**
- [C00.5](https://icdlist.com/icd-10/C00.5) - Malignant neoplasm of lip, unspecified, inner aspect **BILLABLE CODE**
- [C00.6](https://icdlist.com/icd-10/C00.6) - Malignant neoplasm of commissure of lip, unspecified **BILLABLE CODE**
- [C00.8](https://icdlist.com/icd-10/C00.8) - Malignant neoplasm of overlapping sites of lip **BILLABLE CODE**
- [C00.9](https://icdlist.com/icd-10/C00.9) - Malignant neoplasm of lip, unspecified **BILLABLE CODE**

[Malignant neoplasm of base of tongue (C01)](https://icdlist.com/icd-10/index/malignant-neoplasm-of-base-of-tongue-c01)

- [C01](https://icdlist.com/icd-10/C01) - Malignant neoplasm of base of tongue **BILLABLE CODE**

[Malignant neoplasm of other and unspecified parts of tongue (C02)](https://icdlist.com/icd-10/index/malignant-neoplasm-of-other-and-unspecified-parts-of-tongue-c02)

- [C02.0](https://icdlist.com/icd-10/C02.0) - Malignant neoplasm of dorsal surface of tongue **BILLABLE CODE**
- [C02.1](https://icdlist.com/icd-10/C02.1) - Malignant neoplasm of border of tongue **BILLABLE CODE**
- [C02.2](https://icdlist.com/icd-10/C02.2) - Malignant neoplasm of ventral surface of tongue **BILLABLE CODE**
- [C02.3](https://icdlist.com/icd-10/C02.3) - Malig neoplasm of anterior two-thirds of tongue, part unsp **BILLABLE CODE**
- [C02.4](https://icdlist.com/icd-10/C02.4) - Malignant neoplasm of lingual tonsil **BILLABLE CODE**
- [C02.8](https://icdlist.com/icd-10/C02.8) - Malignant neoplasm of overlapping sites of tongue **BILLABLE CODE**
- [C02.9](https://icdlist.com/icd-10/C02.9) - Malignant neoplasm of tongue, unspecified **BILLABLE CODE**

[Malignant neoplasm of gum (C03)](https://icdlist.com/icd-10/index/malignant-neoplasm-of-gum-c03)

- [C03.0](https://icdlist.com/icd-10/C03.0) - Malignant neoplasm of upper gum **BILLABLE CODE**
- [C03.1](https://icdlist.com/icd-10/C03.1) - Malignant neoplasm of lower gum **BILLABLE CODE**
- [C03.9](https://icdlist.com/icd-10/C03.9) - Malignant neoplasm of gum, unspecified **BILLABLE CODE**

[Malignant neoplasm of floor of mouth (C04)](https://icdlist.com/icd-10/index/malignant-neoplasm-of-floor-of-mouth-c04)

- [C04.0](https://icdlist.com/icd-10/C04.0) - Malignant neoplasm of anterior floor of mouth **BILLABLE CODE**
- [C04.1](https://icdlist.com/icd-10/C04.1) - Malignant neoplasm of lateral floor of mouth **BILLABLE CODE**
- [C04.8](https://icdlist.com/icd-10/C04.8) - Malignant neoplasm of overlapping sites of floor of mouth **BILLABLE CODE**
- [C04.9](https://icdlist.com/icd-10/C04.9) - Malignant neoplasm of floor of mouth, unspecified **BILLABLE CODE**

[Malignant neoplasm of palate (C05)](https://icdlist.com/icd-10/index/malignant-neoplasm-of-palate-c05)

- [C05.0](https://icdlist.com/icd-10/C05.0) - Malignant neoplasm of hard palate **BILLABLE CODE**
- [C05.1](https://icdlist.com/icd-10/C05.1) - Malignant neoplasm of soft palate **BILLABLE CODE**
- [C05.2](https://icdlist.com/icd-10/C05.2) - Malignant neoplasm of uvula **BILLABLE CODE**
- [C05.8](https://icdlist.com/icd-10/C05.8) - Malignant neoplasm of overlapping sites of palate **BILLABLE CODE**
- [C05.9](https://icdlist.com/icd-10/C05.9) - Malignant neoplasm of palate, unspecified **BILLABLE CODE**

[Malignant neoplasm of other and unspecified parts of mouth (C06)](https://icdlist.com/icd-10/index/malignant-neoplasm-of-other-and-unspecified-parts-of-mouth-c06)

- [C06.0](https://icdlist.com/icd-10/C06.0) - Malignant neoplasm of cheek mucosa **BILLABLE CODE**
- [C06.1](https://icdlist.com/icd-10/C06.1) - Malignant neoplasm of vestibule of mouth **BILLABLE CODE**
- [C06.2](https://icdlist.com/icd-10/C06.2) - Malignant neoplasm of retromolar area **BILLABLE CODE**
- [C06.80](https://icdlist.com/icd-10/C06.80) - Malignant neoplasm of ovrlp sites of unsp parts of mouth **BILLABLE CODE**
- [C06.89](https://icdlist.com/icd-10/C06.89) - Malignant neoplasm of overlapping sites of oth prt mouth **BILLABLE CODE**
- [C06.9](https://icdlist.com/icd-10/C06.9) - Malignant neoplasm of mouth, unspecified **BILLABLE CODE**

[Malignant neoplasm of parotid gland (C07)](https://icdlist.com/icd-10/index/malignant-neoplasm-of-parotid-gland-c07)

- [C07](https://icdlist.com/icd-10/C07) - Malignant neoplasm of parotid gland **BILLABLE CODE**

[Malignant neoplasm of other and unsp major salivary glands (C08)](https://icdlist.com/icd-10/index/malignant-neoplasm-of-other-and-unsp-major-salivary-glands-c08)

- [C08.0](https://icdlist.com/icd-10/C08.0) - Malignant neoplasm of submandibular gland **BILLABLE CODE**
- [C08.1](https://icdlist.com/icd-10/C08.1) - Malignant neoplasm of sublingual gland **BILLABLE CODE**
- [C08.9](https://icdlist.com/icd-10/C08.9) - Malignant neoplasm of major salivary gland, unspecified **BILLABLE CODE**

[Malignant neoplasm of tonsil (C09)](https://icdlist.com/icd-10/index/malignant-neoplasm-of-tonsil-c09)

- [C09.0](https://icdlist.com/icd-10/C09.0) - Malignant neoplasm of tonsillar fossa **BILLABLE CODE**
- [C09.1](https://icdlist.com/icd-10/C09.1) - Malig neoplasm of tonsillar pillar (anterior) (posterior) **BILLABLE CODE**
- [C09.8](https://icdlist.com/icd-10/C09.8) - Malignant neoplasm of overlapping sites of tonsil **BILLABLE CODE**
- [C09.9](https://icdlist.com/icd-10/C09.9) - Malignant neoplasm of tonsil, unspecified **BILLABLE CODE**

[Malignant neoplasm of oropharynx (C10)](https://icdlist.com/icd-10/index/malignant-neoplasm-of-oropharynx-c10)

- [C10.0](https://icdlist.com/icd-10/C10.0) - Malignant neoplasm of vallecula **BILLABLE CODE**
- [C10.1](https://icdlist.com/icd-10/C10.1) - Malignant neoplasm of anterior surface of epiglottis **BILLABLE CODE**
- [C10.2](https://icdlist.com/icd-10/C10.2) - Malignant neoplasm of lateral wall of oropharynx **BILLABLE CODE**
- [C10.3](https://icdlist.com/icd-10/C10.3) - Malignant neoplasm of posterior wall of oropharynx **BILLABLE CODE**
- [C10.4](https://icdlist.com/icd-10/C10.4) - Malignant neoplasm of branchial cleft **BILLABLE CODE**
- [C10.8](https://icdlist.com/icd-10/C10.8) - Malignant neoplasm of overlapping sites of oropharynx **BILLABLE CODE**
- [C10.9](https://icdlist.com/icd-10/C10.9) - Malignant neoplasm of oropharynx, unspecified **BILLABLE CODE**

[Malignant neoplasm of nasopharynx (C11)](https://icdlist.com/icd-10/index/malignant-neoplasm-of-nasopharynx-c11)

- [C11.0](https://icdlist.com/icd-10/C11.0) - Malignant neoplasm of superior wall of nasopharynx **BILLABLE CODE**
- [C11.1](https://icdlist.com/icd-10/C11.1) - Malignant neoplasm of posterior wall of nasopharynx **BILLABLE CODE**
- [C11.2](https://icdlist.com/icd-10/C11.2) - Malignant neoplasm of lateral wall of nasopharynx **BILLABLE CODE**
- [C11.3](https://icdlist.com/icd-10/C11.3) - Malignant neoplasm of anterior wall of nasopharynx **BILLABLE CODE**
- [C11.8](https://icdlist.com/icd-10/C11.8) - Malignant neoplasm of overlapping sites of nasopharynx **BILLABLE CODE**
- [C11.9](https://icdlist.com/icd-10/C11.9) - Malignant neoplasm of nasopharynx, unspecified **BILLABLE CODE**

[Malignant neoplasm of pyriform sinus (C12)](https://icdlist.com/icd-10/index/malignant-neoplasm-of-pyriform-sinus-c12)

- [C12](https://icdlist.com/icd-10/C12) - Malignant neoplasm of pyriform sinus **BILLABLE CODE**

[Malignant neoplasm of hypopharynx (C13)](https://icdlist.com/icd-10/index/malignant-neoplasm-of-hypopharynx-c13)

- [C13.0](https://icdlist.com/icd-10/C13.0) - Malignant neoplasm of postcricoid region **BILLABLE CODE**
- [C13.1](https://icdlist.com/icd-10/C13.1) - Malig neoplasm of aryepiglottic fold, hypopharyngeal aspect **BILLABLE CODE**
- [C13.2](https://icdlist.com/icd-10/C13.2) - Malignant neoplasm of posterior wall of hypopharynx **BILLABLE CODE**
- [C13.8](https://icdlist.com/icd-10/C13.8) - Malignant neoplasm of overlapping sites of hypopharynx **BILLABLE CODE**
- [C13.9](https://icdlist.com/icd-10/C13.9) - Malignant neoplasm of hypopharynx, unspecified **BILLABLE CODE**

[Malig neoplasm of sites in the lip, oral cavity and pharynx (C14)](https://icdlist.com/icd-10/index/malig-neoplasm-of-sites-in-the-lip-oral-cavity-and-pharynx-c14)

- [C14.0](https://icdlist.com/icd-10/C14.0) - Malignant neoplasm of pharynx, unspecified **BILLABLE CODE**
- [C14.2](https://icdlist.com/icd-10/C14.2) - Malignant neoplasm of Waldeyer's ring **BILLABLE CODE**
- [C14.8](https://icdlist.com/icd-10/C14.8) - Malig neoplm of ovrlp sites of lip, oral cavity and pharynx **BILLABLE CODE**

**Renal cell carcinoma**

C641, **Malignant neoplasm of right kidney, except renal pelvis**

C642, **Malignant neoplasm of left kidney, except renal pelvis**

C649, **Malignant neoplasm of unspecified kidney, except renal pelvis**

**Opioid Dependence**

F1120
