# Supplementary material for: Disparities and burden of illness among hospitalized cancer patients with opioid dependence
Source: Med Oncol. 2026 Sep 5;43(10):267. doi: 10.1007/s12032-026-03371-x (PMC13546320; doi:10.1007/s12032-026-03371-x)
Supplement: Supplementary file 2 [file 12032_2026_3371_MOESM2_ESM.docx]

Supplementary file 2.

Table. Multivariable Generalized Linear Model of Depressive disorders among PC, RCC, and CLOP patients. Adjusted odds ratio of having depressive disorders for patients with opioid dependency when compared to non-dependent patients with adjusted factors.

| **DEPRESSIVE DISORDERS** | **Prostate Cancer Adjusted Odds Ratio** | **Renal Cell Carcinoma Adjusted Odds Ratio** | **Cancers of the Lip, Oral Cavity, and Pharynx Adjusted Odds Ratio** |
| --- | --- | --- | --- |
| Non-Opioid Dependent Cancer Patients | **Reference** | **Reference** | **Reference** |
| Opioid-dependent Cancer Patients | 2.22 (1.48, 3.33) | 0.55 (0.32, 0.94) | 1.28 (0.76, 2.16) |
|  |  |  |  |
| **Age** | 0.99 (0.98, 0.99) | 1.01 (1.01, 1.02) | 0.98 (0.97, 0.99) |
| **Gender (Male)** |  | **Reference** | **Reference** |
| Female |  | 0.62 (0.57, 0.67) | 1.40 (1.23, 1.59) |
| **Admission type** |  |  |  |
| Non-elective | **Reference** | **Reference** | **Reference** |
| Elective | 0.42 (0.38, 0.48) | 1.07 (0.98, 1.17) | 0.65 (0.56, 0.75) |
| **Expected primary payer** |  |  |  |
| Medicare | **Reference** | **Reference** | **Reference** |
| Medicaid | 1.08 (0.89, 1.29) | 0.84 (0.71, 0.98) | 0.86 (0.69, 1.06) |
| Private Insurance | 0.64 (0.57, 0.73) | 0.90 (0.82, 1.00) | 0.72 (0.60, 0.86) |
| Self-pay & No Charge & Other | 0.83 (0.66, 1.03) | 0.95 (0.78, 1.15) | 0.58 (0.41, 0.82) |
| **Patient Location: NCHS Urban-Rural Code** |  |  |  |
| Central counties of metro areas of >= 1 million population | **Reference** | **Reference** | **Reference** |
| Fringe counties of metro areas of >= 1 million population | 0.92 (0.82, 1.03) | 1.06 (0.95, 1.19) | 0.94 (0.78, 1.13) |
| Counties in metro areas of 250,000-999,999 population | 0.96 (0.85, 1.08) | 1.03 (0.91, 1.16) | 1.00 (0.82, 1.21) |
| Counties in metro areas of 50,000-249,999 population | 0.86 (0.74, 1.01) | 1.07 (0.92, 1.24) | 1.29 (0.99, 1.68) |
| Micropolitan counties & Not metropolitan or micropolitan counties | 0.91 (0.80, 1.04) | 0.99 (0.87, 1.13) | 1.03 (0.83, 1.29) |
| **Racial and Ethnic Characteristics** |  |  |  |
| White | **Reference** | **Reference** | **Reference** |
| Black | 0.58 (0.51, 0.66) | 0.90 (0.79, 1.01) | 0.66 (0.52, 0.83) |
| Hispanic | 0.62 (0.52, 0.74) | 0.82 (0.71, 0.95) | 0.74 (0.56, 0.97) |
| Asian and others | 0.57 (0.47, 0.69) | 0.83 (0.69, 1.00) | 0.48 (0.36, 0.65) |
| **Median household income (based on current year)** |  |  |  |
| 76^th^ to 100^th^ percentile | **Reference** | **Reference** | **Reference** |
| 0-25^th^ percentile | 0.98 (0.86, 1.12) | 0.99 (0.87, 1.12) | 1.03 (0.84, 1.26) |
| 26^th^ to 50^th^ percentile | 0.99 (0.88, 1.12) | 1.04 (0.93, 1.16) | 0.93 (0.76, 1.14) |
| 51^st^ to 75^th^ percentile | 1.04 (0.93, 1.15) | 1.05 (0.94, 1.17) | 0.92 (0.76, 1.12) |
| **Weighted Elixir Score Mean** | 0.97 (0.97, 0.98) | 1.00 (1.00, 1.01) | 0.97 (0.96, 0.98) |
