# Supplementary material for: Disparities and burden of illness among hospitalized cancer patients with opioid dependence
Source: Med Oncol. 2026 Sep 5;43(10):267. doi: 10.1007/s12032-026-03371-x (PMC13546320; doi:10.1007/s12032-026-03371-x)
Supplement: Supplementary file 3 [file 12032_2026_3371_MOESM3_ESM.docx]

Supplementary file 3

Table. Multivariable Generalized Linear Model of Anxiety Disorders among PC, RCC, and CLOP patients. Adjusted odds ratio of having anxiety disorders for patients with opioid dependency when compared to non-dependent patients with adjusted factors.

| **ANXIETY DISORDERS** | **Adjusted Odds Ratio** | **Renal Cell Carcinoma Adjusted Odds Ratio** | **Cancers of the Lip, Oral Cavity, and Pharynx Adjusted Odds Ratio** |
| --- | --- | --- | --- |
| Non-Opioid Dependent Cancer Patients | **Reference** | **Reference** | **Reference** |
| Opioid-dependent Cancer Patients | 2.31 (1.53, 3.48) | 2.10 (1.31, 3.38) | 1.94 (1.22, 3.08) |
|  |  |  |  |
| **Age** | 0.97 (0.96, 0.97) | 0.97 (0.97, 0.98) | 0.97 (0.97, 0.98) |
| **Gender (Male)** |  | **Reference** | **Reference** |
| Female |  | 2.01 (1.83, 2.21) | 1.86 (1.65 (2.09) |
| **Admission type** |  |  |  |
| Non-elective | **Reference** | **Reference** | **Reference** |
| Elective | 0.61 (0.54, 0.68) | 0.79 (0.70, 0.90) | 0.83 (0.73, 0.94) |
| **Expected primary payer** |  |  |  |
| Medicare | **Reference** | **Reference** | **Reference** |
| Medicaid | 0.90 (0.74, 1.09) | 0.94 (0.77, 1.14) | 0.85 (0.69, 1.04) |
| Private Insurance | 0.70 (0.62, 0.78) | 0.77 (0.67, 0.89) | 0.91 (0.77, 1.07) |
| Self-pay & No Charge & Other | 0.81 (0.65, 1.01) | 0.74 (0.58, 0.96) | 0.80 (0.60, 1.07) |
| **Patient Location: NCHS Urban-Rural Code** |  |  |  |
| Central counties of metro areas of >= 1 million population | **Reference** | **Reference** | **Reference** |
| Fringe counties of metro areas of >= 1 million population | 0.99 (0.89, 1.10) | 1.12 (0.98, 1.29) | 1.12 (0.95, 1.31) |
| Counties in metro areas of 250,000-999,999 population | 1.01 (0.89, 1.14) | 0.99 (0.85, 1.14) | 1.22 (1.02, 1.46) |
| Counties in metro areas of 50,000-249,999 population | 0.96 (0.82, 1.12) | 1.11 (0.92, 1.35) | 1.17 (0.92, 1.49) |
| Micropolitan counties & Not metropolitan or micropolitan counties | 0.97 (0.85, 1.10) | 1.05 (0.89, 1.23) | 1.07 (0.87, 1.32) |
| **Racial and Ethnic Characteristics** |  |  |  |
| White | **Reference** | **Reference** | **Reference** |
| Black | 0.43 (0.34, 0.49) | 0.49 (0.40, 0.59) | 0.44 (0.35, 0.56) |
| Hispanic | 0.59 (0.49, 0.70) | 0.51 (0.42, 0.62) | 0.66 (0.51, 0.86) |
| Asian and others | 0.64 (0.50, 0.81) | 0.54 (0.40, 0.74) | 0.55 (0.38, 0.78) |
| **Median household income (based on current year)** |  |  |  |
| 76^th^ to 100^th^ percentile | **Reference** | **Reference** | **Reference** |
| 0-25^th^ percentile | 1.02 (0.90, 1.16) | 1.09 (0.93, 1.29) | 1.02 (0.85, 1.22) |
| 26^th^ to 50^th^ percentile | 1.06 (0.94, 1.19) | 1.04 (0.89, 1.21) | 1.00 (0.84, 1.19) |
| 51^st^ to 75^th^ percentile | 1.06 (0.94, 1.18) | 1.06 (0.91, 1.23) | 0.90 (0.76, 1.07) |
| **Weighted Elixir Score Mean** | 1.00 (1.00, 1.00) | 1.00 (0.99, 1.00) | 1.00 (0.99, 1.01) |
