# Supplementary material for: Disparities and burden of illness among hospitalized cancer patients with opioid dependence
Source: Med Oncol. 2026 Sep 5;43(10):267. doi: 10.1007/s12032-026-03371-x (PMC13546320; doi:10.1007/s12032-026-03371-x)
Supplement: Supplementary file 5 [file 12032_2026_3371_MOESM5_ESM.docx]

Supplementary file 5

Table. Multivariable Generalized Linear Model of weight loss among PC, RCC, and CLOP patients. Adjusted odds ratio of weight loss for patients with opioid dependency when compared to non-dependent patients with adjusted factors.

| **Weight Loss** | **Prostate Adjusted Odds Ratio** | **Renal cell carcinoma Adjusted Odds Ratio** | **Cancers of the Lip, Oral Cavity, and Pharynx Adjusted Odds Ratio** |
| --- | --- | --- | --- |
| Non-Opioid Dependent Cancer Patients | **Reference** | **Reference** | **Reference** |
| Opioid-dependent Cancer Patients | 2.96 (1.81, 4.83) | 1.46 (0.71, 2.99) | 2.21 (1.36, 3.59) |
|  |  |  |  |
| **Age** | 1.00 (0.99, 1.00) | 1.00 (0.99, 1.00) | 0.99 (0.98, 0.99) |
| **Gender (Male)** |  | **Reference** | **Reference** |
| Female |  | 1.18 (1.05, 1.33) | 1.06 (0.95, 0.59) |
| **Admission type** |  |  |  |
| Non-elective | **Reference** | **Reference** | **Reference** |
| Elective | 0.49 (0.42, 0.57) | 0.53 (0.45, 0.62) | 0.52 (0.46, 0.59) |
| **Expected primary payer** |  |  |  |
| Medicare | **Reference** | **Reference** | **Reference** |
| Medicaid | 1.20 (1.01, 1.43) | 1.59 (1.29, 1.97) | 1.49 (1.26, 1.76) |
| Private Insurance | 0.85 (0.75, 0.96) | 1.15 (0.98, 1.35) | 0.97 (0.85, 1.11) |
| Self-pay & No Charge & Other | 1.09 (0.88, 1.32) | 1.44 (1.09, 1.89) | 1.02 (0.80, 1.30) |
| **Patient Location: NCHS Urban-Rural Code** |  |  |  |
| Central counties of metro areas of >= 1 million population | **Reference** | **Reference** | **Reference** |
| Fringe counties of metro areas of >= 1 million population | 0.89 (0.79, 1.00) | 0.97 (0.82, 1.15) | 0.99 (0.86, 1.15) |
| Counties in metro areas of 250,000-999,999 population | 0.87 (0.77, 0.99) | 0.96 (0.80, 1.15) | 0.99 (0.85, 1.15) |
| Counties in metro areas of 50,000-249,999 population | 0.87 (0.72, 1.04) | 0.94 (0.74, 1.19) | 0.95 (0.78, 1.15) |
| Micropolitan counties & Not metropolitan or micropolitan counties | 0.87 (0.75, 1.00) | 1.05 (0.86, 1.28) | 0.93 (0.78, 1.11) |
| **Racial and Ethnic Characteristics** |  |  |  |
| White | **Reference** | **Reference** | **Reference** |
| Black | 1.40 (1.25, 1.56) | 1.03 (0.86, 1.24) | 1.22 (1.04, 1.43) |
| Hispanic | 1.12 (0.96, 1.32) | 0.96 (0.79, 1.18) | 0.95 (0.77, 1.16) |
| Asian and others | 1.35 (1.15, 1.58) | 1.19 (0.92, 1.55) | 0.78 (0.60, 1.00) |
| **Median household income (based on current year)** |  |  |  |
| 76^th^ to 100^th^ percentile | **Reference** | **Reference** | **Reference** |
| 0-25^th^ percentile | 1.10 (0.97, 1.25) | 1.08 (0.89, 1.32) | 1.20 (1.02, 1.40) |
| 26^th^ to 50^th^ percentile | 1.04 (0.91, 1.18) | 1.10 (0.92, 1.32) | 1.32 (1.12, 1.54) |
| 51^st^ to 75^th^ percentile | 0.96 (0.86, 1.09) | 1.16 (0.98, 1.40) | 1.12 (0.97, 1.32) |
| **Weighted Elixir Score Mean** | 1.12 (1.11, 1.12) | 1.13 (1.12, 1.13) | 1.11 (1.10, 1.12) |
