# Supplementary material for: Disparities and burden of illness among hospitalized cancer patients with opioid dependence
Source: Med Oncol. 2026 Sep 5;43(10):267. doi: 10.1007/s12032-026-03371-x (PMC13546320; doi:10.1007/s12032-026-03371-x)
Supplement: Supplementary file 6 [file 12032_2026_3371_MOESM6_ESM.docx]

Supplementary file 6

Table. Multivariable Generalized Linear Model of emergency admission among PC, RCC, and CLOP patients. Adjusted odds ratio of emergency admission for patients with opioid dependency when compared to non-dependent patients with adjusted factors.

| **Emergency Admissions** | **Prostate Adjusted Odds Ratio** | **Renal cell carcinoma Adjusted Odds Ratio** | **Cancers of the Lip, Oral Cavity, and Pharynx Adjusted Odds Ratio** |
| --- | --- | --- | --- |
| Non-Opioid Dependent Cancer Patients | **Reference** | **Reference** | **Reference** |
| Opioid-dependent Cancer Patients | 2.60 (1.47, 4.59) | 1.66 (1.00, 2.73) | 1.21 (0.68, 2.16) |
|  |  |  |  |
| **Age** | 1.02 (1.02, 1.03) | 1.01 (1.01, 1.02) | 1.01 (1.00, 1.01) |
| **Gender (Male)** |  | **Reference** | **Reference** |
| Female |  | 1.12 (1.01, 1.25) | 0.98 (0.86, 1.12) |
| **Admission type** |  |  |  |
| Non-elective | **Reference** | **Reference** | **Reference** |
| Elective | 0.01 (0.01, 0.01) | 0.01 (0.00, 0.01) | 0.01 (0.01, 0.01) |
| **Expected primary payer** |  |  |  |
| Medicare | **Reference** | **Reference** | **Reference** |
| Medicaid | 1.20 (0.98, 1.47) | 1.19 (0.96, 1.46) | 0.96 (0.79, 1.16) |
| Private Insurance | 0.81 (0.72, 0.92) | 0.86 (0.74, 0.99) | 0.78 (0.66, 0.92) |
| Self-pay & No Charge & Other | 0.90 (0.74, 1.08) | 0.91 (0.68, 1.22) | 1.21 (0.90, 1.62) |
| **Patient Location: NCHS Urban-Rural Code** |  |  |  |
| Central counties of metro areas of >= 1 million population | **Reference** | **Reference** | **Reference** |
| Fringe counties of metro areas of >= 1 million population | 0.84 (0.71, 1.00) | 0.85 (0.69, 1.04) | 1.02 (0.83, 1.26) |
| Counties in metro areas of 250,000-999,999 population | 0.96 (0.88, 1.19) | 0.89 (0.71, 1.12) | 1.06 (0.85, 1.32) |
| Counties in metro areas of 50,000-249,999 population | 0.87 (0.69, 1.10) | 0.75 (0.56, 1.00) | 1.11 (0.85, 1.47) |
| Micropolitan counties & Not metropolitan or micropolitan counties | 0.42 (0.35, 0.51) | 0.46 (0.37, 0.57) | 0.67 (0.53, 0.85) |
| **Racial and Ethnic Characteristics** |  |  |  |
| White | **Reference** | **Reference** | **Reference** |
| Black | 1.13 (0.95, 1.34) | 1.10 (0.90, 1.34) | 1.20 (0.96, 1.49) |
| Hispanic | 1.49 (1.24, 1.80) | 1.17 (0.95, 1.45) | 1.63 (1.25, 2.12) |
| Asian and others | 1.11 (0.92, 1.33) | 1.02 (0.78, 1.32) | 1.16 (0.89, 1.52) |
| **Median household income (based on current year)** |  |  |  |
| 76^th^ to 100^th^ percentile | **Reference** | **Reference** | **Reference** |
| 0-25^th^ percentile | 0.84 (0.71, 0.98) | 1.01 (0.83, 1.23) | 0.91 (0.72, 1.15) |
| 26^th^ to 50^th^ percentile | 0.76 (0.66, 0.88) | 0.98 (0.82, 1.18) | 0.81 (0.66, 0.97) |
| 51^st^ to 75^th^ percentile | 0.82 (0.72, 0.94) | 0.90 (0.76, 1.06) | 0.85 (0.70, 1.02) |
| **Weighted Elixir Score Mean** | 1.02 (1.02, 1.03) | 1.02 (1.02, 1.03) | 1.01 (1.01, 1.02) |
